# Short-Term Outcomes of Cataract Surgery in Patients with a History of Central Serous Chorioretinopathy

**DOI:** 10.1155/2021/9952050

**Published:** 2021-06-24

**Authors:** Jeon Young Joon, Jae Hui Kim, Jong Woo Kim, Chul Gu Kim

**Affiliations:** Department of Ophthalmology, Kim's Eye Hospital, Konyang University College of Medicine, Seoul, Republic of Korea

## Abstract

**Purpose:**

This study aimed to evaluate the short-term outcomes of cataract surgery in patients with a history of idiopathic central serous chorioretinopathy (CSC).

**Methods:**

This retrospective study included 26 patients with a history of CSC who underwent uncomplicated phacoemulsification and intraocular lens implantation. The best-corrected visual acuity (BCVA) and central foveal thickness (CFT) measured before the surgery were compared with those obtained at mean 3.6 months after the surgery. BCVA outcome was also analyzed in eyes with and without subretinal fluid (SRF).

**Results:**

The mean logarithm of the minimal angle of resolution BCVA significantly improved from 0.52 ± 0.40 before the surgery to 0.21 ± 0.30 one month after the surgery and 0.20 ± 0.31 at 3.6 months after the surgery (*P* < 0.001). The mean CFT was 281.2 ± 84.2 *μ*m before the surgery, 301.5 ± 90.7 *μ*m one month after the surgery, and 279.9 ± 83.6 *μ*m at 3.6 months after the surgery. The CFT before surgery was not different from those measured at 3 months (*P*=0.253). Significant improvement in BCVA at 3.6 months was noted in the SRF group (*N* = 12, *P*=0.003) and the non-SRF group (*N* = 14, *P*=0.001). CSC recurrence was noted in 2 patients in the non-SRF group.

**Conclusions:**

Significant improvement in visual acuity was noted after cataract surgery in patients with a history of idiopathic CSC, without a risk of aggravation of CSC in most patients.

## 1. Introduction

Central serous chorioretinopathy (CSC) is a disorder that is characterized by a localized accumulation of subretinal fluid (SRF) in the macula due to a leakage from the retinal pigment epithelium (RPE) [1]. It usually develops in young men [2]. Additionally, the incidence of CSC is higher in Asians than in Caucasians [2]. Although idiopathic CSC is often self-limiting, [3, 4] chronic CSC can be a sight-threatening disease [5, 6].

The exact etiology of CSC is not clearly understood. However, investigators have postulated that choroidal pathophysiology has a potential role in its etiology [7]. In fact, choroidal thickness (CT) in patients with CSC is markedly greater than that of normal subjects [8] or uninvolved fellow eyes [9]. Additionally, eyes with CSC frequently exhibit choroidal vascular hyperpermeability [10], larger choroidal vessel diameter, [11] and abnormal choroidal vascular reactivity [12], suggesting a dysregulated choroidal blood flow.

Phacoemulsification is currently the standard of care for cataract removal. Although phacoemulsification and implantation of intraocular lens is a safe and effective method [13], it is reported that this surgery can affect the choroid [14–17]. Since CT is associated with CSC activity [18–21], the outcome of cataract surgery in patients with CSC can be influenced by the impact of the surgery on the choroid. To date, however, the outcomes of cataract surgery in patients with CSC have not been fully elucidated.

The present study aimed to evaluate the short-term outcomes of uncomplicated cataract surgery in patients with a history of idiopathic CSC.

## 2. Materials and Methods

The present retrospective observational study was conducted in a single center. The study was approved by the Institutional Review Board of Kim's Eye Hospital and was conducted in accordance with the tenets of the Declaration of Helsinki. Due to the retrospective nature of this study, the need for informed consent was waived off (Kim's Eye Hospital IRB, Seoul, South Korea).

### 2.1. Patients

Among the patients who had been diagnosed with idiopathic CSC at Kim's Eye Hospital between January 2011 and September 2019, patients undergoing uncomplicated cataract surgery using phacoemulsification were included. Patients with active CSC and patients with a previous history of CSC without SRF before cataract surgery were included. The exclusion criteria were as follows: (1) less than 3 months of follow-up, (2) retinal disorders other than CSC that may influence macular structure or function, (3) history of uveitis, (4) evidence of choroidal neovascularization, and (5) history of vitreoretinal surgery.

### 2.2. Examinations

At diagnosis, ophthalmological examinations, including measurement of best-corrected visual acuity (BCVA), 90D lens slit-lamp biomicroscopy, fundus photography, and fluorescein angiography, were performed in all patients. Additionally, optical coherence tomography (OCT) images were obtained using any of the following: Spectralis® (Heidelberg Engineering GmbH, Germany), RS 3000® (Nidek Co., Ltd., Tokyo, Japan), or Spectral OCT/SLO® (OTI Ophthalmic Technologies Inc., Toronto, Canada).

Right after establishing the initial diagnosis of CSC, patients were closely observed without active treatment. In cases where SRF persisted more than 4 months, focal laser photocoagulation or intravitreal bevacizumab injection was performed in selected patients at the discretion of the treating physician. When patients exhibited extrafoveal focal leakage on fluorescein angiography, the patients were treated with focal laser photocoagulation. When subfoveal focal leakage or diffuse leakage was noted on fluorescein angiography, bevacizumab injection was performed.

### 2.3. Cataract Surgery and Follow-Up

Cataract surgery was planned when the treating physician determined its necessity to restore vision. The cataract was graded using the Lens Opacity Classification System III in selected cases. BCVA was measured 1–4 weeks before the surgery. Simultaneously, OCT examination was performed to identify the presence of SRF, and axial length was measured using IOL Master® (Carl Zeiss Meditec AG, Jena, Germany). Cataract surgery was performed using a small incision (2.8 mm to 3.0 mm) phacoemulsification and implantation of a foldable intraocular lens. After the surgery, patients were treated with topical antibiotics and topical steroids for 1 month. BCVA measurement and OCT examination were performed at 1 month and 3–5 months (mean, 3.6 ± 0.8 months) after the surgery.

### 2.4. Outcome Measures

For all the included eyes, the BCVA and CFT values measured before cataract surgery were compared with those measured at 3.6 months after surgery. Eyes were divided into 2 groups according to the presence of SRF before the surgery: the SRF group versus the non-SRF group. Within each group, BCVA and CFT values measured before cataract surgery were compared with those measured at 3.7 months (SRF group) or 3.5 months (non-SRF group) after the surgery. In the non-SRF group, the rate of CSC recurrence was also identified.

BCVAs were measured using the decimal visual acuity chart and converted to logarithm of a minimum angle of resolution (logMAR) values for analysis. Central foveal thickness (CFT) was defined as the vertical distance between Bruch's membrane and the internal limiting membrane. The CFT was manually measured based on OCT images using image J program (National Institutes of Health, Bethesda, Maryland, USA).

### 2.5. Statistical Analyses

The statistical analysis methods used in this study were similar to those in our previous study [22]. Statistical analyses were performed using SPSS program (version 12.0, International Business Machines Corporation, Armonk, NY, USA). Shapiro-Wilk test was performed to identify normal distribution. Values between different time points were compared using the Wilcoxon signed-ranks test. Values between different groups were compared using the Mann-Whitney *U* test and Fisher's exact test with a Bonferroni's correction. Binary logistic regression was used to analyze factors associated with a change in BCVA. *P* values less than 0.05 were considered statistically significant.

## 3. Results

**Figure 1:**
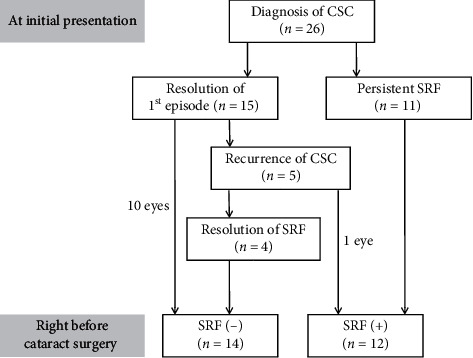
A diagram showing the clinical course of the included patients between the initial diagnosis of central serous chorioretinopathy and cataract surgery. SRF = subretinal fluid, CSC = central serous chorioretinopathy.

Twenty-six eyes from 26 patients (19 men and 7 women) were included in the study (Table 1). Regarding CSC treatment, 4 eyes underwent focal laser photocoagulation, 5 eyes underwent intravitreal bevacizumab injection, and one eye underwent both focal laser photocoagulation and bevacizumab injection. The remaining 16 eyes were followed up without treatment. The first episode of CSC completely resolved in 15 eyes (CSC recurred in 5 of them), whereas SRF did not completely resolve until the cataract surgery was performed in 11 eyes. Among the 15 eyes with a resolution of the first episode, CSC recurred in 5 eyes. On examination before cataract surgery, SRF was not noted in 14 eyes. In these eyes, the mean fluid-free period was 35.5 ± 28.3 months (mean ± standard deviation) before the surgery.  Figure 1 shows the clinical course of the included patients between the initial diagnosis of CSC and cataract surgery.

Grading of cataracts was performed in 12 patients (46.2%) who had either NO2 or a higher degree of nuclear opacity, C3 or higher degree of cortical opacity, or P2 or higher degree of posterior subcapsular opacity. In the remaining 14 patients (53.8%), the accurate degree of cataract was not identified. Cataract surgery was performed at 32.8 ± 33.6 months after CSC initial diagnosis. The mean surgery time, from incision to wound closure, was 12.0 ± 2.5 minutes. All the surgeries were successfully performed without any intraoperative or postoperative complications, such as posterior capsular rupture, endophthalmitis, or development of pseudophakic cystoid macular edema.

The mean CFTs were 281.2 ± 84.2 *μ*m before the surgery, 301.5 ± 90.7 *μ*m 1 month after the surgery, and 279.9 ± 83.6 *μ*m at mean 3.6 ± 0.8 months after the surgery. In 17 (65.4%) of 26 patients, OCT scans were performed using a single OCT device for all the three time points (before the surgery, 1 month after surgery, and mean 3.6 months after the surgery). In the remaining 9 patients (34.6%), at least two different OCT devices were used at the three time points. The CFT before surgery was not different from those measured at mean 3.6 months (*P*=0.253) (Table 2). The mean logMAR BCVA was 0.52 ± 0.40 before the surgery, 0.21 ± 0.30 one month after the surgery, and 0.20 ± 0.31 at mean 3.6 after the surgery. When compared to the values before the surgery, BCVA had significantly improved at 3.6 months (*P* < 0.001) (Table 3). Two lines or greater improvement in BCVA was noted in 17 eyes (62.9%) at both 1 month and mean 3.6 months. Deterioration in BCVA was noted in none of the included eyes.

When divided into 2 groups, according to the presence of SRF before the surgery, 12 eyes (46.2%) were included in the SRF group, and the remaining 14 eyes (53.8%) were included in the non-SRF group. In the SRF group, the mean CFTs were 348.6 ± 73.7 *μ*m before the surgery, 361.0 ± 50.5 *μ*m one month after the surgery, and 324.8 ± 78.6 *μ*m at mean 3.7 ± 0.8 months after the surgery. CFTs at 3 months were not different from the values before the surgery (*P*=0.875) (Table 2). At 1 month and at 3 months, a 50 *μ*m or greater increase in CFT was noted in 2 eyes (16.7%) and 1 eye (8.3%), respectively. The mean logMAR BCVAs were 0.72 ± 0.44 before the surgery, 0.34 ± 0.39 1 month after the surgery, and 0.33 ± 0.39 at mean 3.7 ± 0.8 months after the surgery. When compared to the values before surgery, BCVA had significantly improved at 3.6 months (*P*=0.003) (Table 3).

In the non-SRF group, the mean CFTs were 223.5 ± 35.3 *μ*m before the surgery, 250.6 ± 87.3 *μ*m 1 month after the surgery, and 241.5 ± 69.0 *μ*m at mean 3.5 ± 0.8 months after the surgery. CFT at 3 months was not different from the values before the surgery (*P*=0.132) (Table 2). The mean logMAR BCVAs were 0.34 ± 0.26 before the surgery, 0.09 ± 0.13 1 month after the surgery, and 0.10 ± 0.15 at mean 3.5 ± 0.8 months after the surgery. When compared to the values before surgery, BCVA had significantly improved at 3.5 months (*P*=0.001) (Table 3).

**Figure 2:**
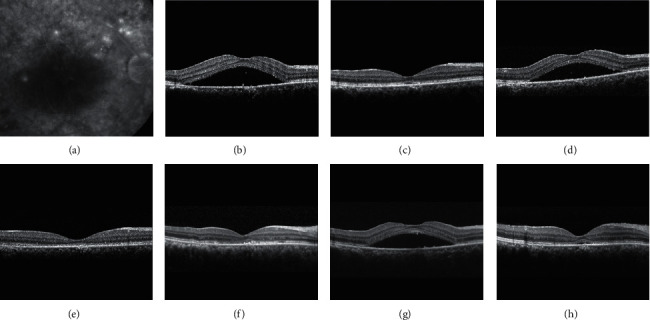
Fluorescein angiography (a) and optical coherence tomography (b–h) images of a 44-year-old patient. Central serous chorioretinopathy (CSC) was first diagnosed 73 months before the cataract surgery (a, b). The best-corrected visual acuity (BCVA) was 0.5. Four months after the diagnosis of CSC, subretinal fluid (SRF) spontaneously resolved without treatment (c). Six months later (d), SRF recurred, and the patient was treated with focal laser photocoagulation. The SRF completely resolved 1 month after treatment (e). The macula remained dry until 1 month before cataract surgery (61 months after the diagnosis of CSC) (f). The BCVA was 0.4 at this time. One month after the surgery, recurrence of CSC was noted and the BCVA was measured as 0.7 (g), but it spontaneously resolved after 2 months with the improvement of BCVA to 0.8 (h).

During the study, recurrence of CSC was noted in 2 eyes (14.3%): one at 1 month and the other one at 3 months after the surgery. In an eye with recurred CSC at 1 month, CSC spontaneously resolved at 3 months (Figure 2). The BCVA in this eye was 0.4 before the surgery, 0.7 one month after the surgery, and 0.8 three months after the surgery. In the other eye with recurred CSC at 3 months, the BCVA was 0.3 before the surgery, 0.7 one month after the surgery, and 0.5 three months after the surgery. At 1 month and at 3 months, a 50 *μ*m or greater increase in CFT was noted in 1 eye (7.1%) each. All the increase in CFT in the 2 eyes were due to recurred CSC. Figure 2 shows a case of recurrence of CSC after the surgery.

## 4. Discussion

CSC usually occurs in young and middle-aged men [2, 23]. In a study by Tsai et al., the annual incidence of CSC was between 0.30% and 0.44% in men aged between 35 and 54 years, whereas the incidence was only between 0.11% and 0.17% in women of the same age group [23]. However, this sex-specific trend changed at the second peak in more aged subjects. In subjects aged 55–59 years, the annual incidence was similar between men (0.21%) and women (0.20%), [23] suggesting that CSC is not a rare disorder in older women. For this reason, investigating the outcome of cataract surgery in both male and female CSC patients is important.

Previous studies have reported an increase in CT after cataract surgery. In the prospective study of Ohsugi et al., CT increased after surgery in the entire macular region, including the subfoveal, nasal, temporal, superior, and inferior regions [16]. The increased CT was maintained for up to 6 months in several regions. Pierru et al. [14] demonstrated a significant increase in subfoveal CT even on day 1 after the surgery, and increased thickness was maintained up to month 3. In the study by Noda et al., the CT significantly increased at 1, 3, and 6 months after the surgery [15]. In 31.0% of the patients, CT returned to normal values at 3 or 6 months. However, CT at 6 months was still thicker than the baseline value in the remaining 69.0% of the patients [24].

Although the reason of this choroidal change after cataract surgery has not yet been clearly elucidated, several investigators suggested potential explanations. Pierru et al. postulated that the release and accumulation of inflammatory mediators, such as prostaglandins, endotoxin, immune complex, and cytokines, due to surgical trauma may induce posterior segment inflammation [14]. Noda et al. postulated that metabolic activation in the RPE due to light exposure during the surgery has a possible influence on the choroidal change [15].

Since the pathogenesis and activity of CSC are closely associated with choroidal thickening, we evaluated whether cataract surgery is safe and effective in patients with a history of CSC. As a result, significant visual improvement was noted after the surgery, and no patient experienced visual deterioration. In the present study, an approximately 20 *μ*m mean increase in CFT was significantly noted at 1 month after cataract surgery. A previous study has shown an increase in retinal thickness of approximately 21.7 *μ*m after cataract surgery in healthy eyes [25]. Thus, a 20 *μ*m increase in CFT in our patient may not be an unusual finding. Additionally, CFT at 3 months was not different from those measured before the surgery.

CSC is often self-limiting, and a spontaneous resolution of SRF occurs within several months in the majority of patients [3, 4]. Thus, physicians usually prefer observation in acute CSC cases [26]. However, a more active approach is required in selected cases to resolve symptoms and to prevent visual deterioration. To date, various treatment modalities including laser photocoagulation, [27] photodynamic therapy, [28] and selective retina therapy [29] have been performed to treat CSC. Although these treatments have notable benefits, they also have limitations and do not guarantee a complete resolution of SRF. In addition, antivascular endothelial growth factor therapy is usually not effective, except in patients with neovascular CSC [30, 31]. Currently, there is no gold standard method used to manage CSC.

When treating CSC patients, it is sometimes difficult for SRF to completely resolve. If the patient also presents cataract progression, cataract surgery is required regardless of the fluid status. In this situation, one concern is whether CSC aggravates after cataract surgery. If CSC severely aggravates after the surgery, it has a significantly negative influence on visual recovery. Thus, the possibility of disease aggravation and recurrence should be discussed with patients before determining cataract surgery. Nevertheless, no previous study has focused on this issue. In the present study, significant visual improvement was noted after cataract surgery even in eyes with SRF. In eyes with SRF, a slight increase in retinal thickness was noted at 1 month, and it was not significantly different from the values before surgery. Additionally, only 1 of the 12 eyes exhibited a 50 *μ*m or greater increase in CFT at 3 months. In eyes without SRF, significant improvement in visual acuity was also noted. Recurrence of CSC was noted in 14.3% of the eyes. However, one of them spontaneously resolved at 3 months. As a result, a 50 *μ*m or greater increase in CFT at 3 months was noted in only 1 of the 14 eyes. These results suggest that cataract surgery can be performed without risk of aggravation of CSC in most patients.

The strength of the present study is that we first focused on the outcomes of cataract surgery in eyes with a history of CSC. However, this study has several notable limitations. First, it was retrospective. Second, the sample size was small. In the present study, the main outcome variables, BCVA and CFT, did not follow a normal distribution and the analysis was performed using nonparametric tests. We postulate that the small sample size may influence this. Third, only short-term outcomes were reported. Fourth, enhanced depth imaging was not routinely performed; therefore, a change in CT was not evaluated. Thus, any association between CT and the activity of CSC could not be investigated. Since choroidal pathophysiology has an important role in the etiology of CSC, the lack of CT data is a major drawback in this study. Fifth, there was no strict guideline to determine cataract surgery. In addition, a standardized grading of cataract was performed only in 46.2% of the patients, thus making it not possible to fully identify how a visually significant cataract was determined. Finally, different OCT devices were used in 9 patients when measuring CFT at different time points. As the measurement of retinal thickness between different OCT devices could vary, the CFT measurements in these 9 patients may have been affected.

In conclusion, significant improvement in visual acuity was noted after uncomplicated cataract surgery in eyes with a history of idiopathic CSC, regardless of the presence of SRF. A definite increase in retinal thickness or recurrence of CSC was noted in only a limited proportion of the patients. Further studies are required to identify whether cataract surgery has a long-term influence on the clinical course of CSC.

## Figures and Tables

**Table 1:** Characteristics of the study population (*n* = 26).

Characteristics	
Age (years)	59.0 ± 0.8
Gender (male: female)	19 (73.1%): 7 (26.9%)
Hypertension	12 (46.2%)
Diabetes mellitus	5 (19.2%)
Axial length (*μ*m)	23.4 ± 1.3

*Type of cataract*	
NS or CO	12 (46.2%)
ASC or PSC with or without NS or CO	14 (53.8%)
Duration between the diagnosis of CSC and cataract surgery (months)	32.8 ± 33.6
Presence of SRF before cataract surgery	12 (46.2%)
Surgery time (minutes)	12.0 ± 2.5

The data are presented as mean ± standard deviation or no. (%) when applicable. NS = nucleosclerosis, CO = cortical opacity, ASC = anterior subcapsular opacity, PSC = posterior subcapsular opacity, CSC = central serous chorioretinopathy, SRF = subretinal fluid.

**Table 2:** Comparison of central foveal thickness before and after cataract surgery.

Groups	Before cataract surgery	After cataract surgery^*∗*^	*P* value^†^
All the included eyes (*n* = 26)	281.2 ± 84.2	279.9 ± 83.6	0.253
SRF group (*n* = 12)	348.6 ± 73.7	324.8 ± 78.6	0.875
Non-SRF group (*n* = 14)	223.5 ± 35.3	241.5 ± 69.0	0.132

The data are presented as mean ± standard deviation. SRF = subretinal fluid. ^*∗*^The timing of measurement was averagely 3.6 months in all the included eyes, 3.7 months in the SRF group, and 3.5 months in the non-SRF group. ^†^Statistical analysis was performed using Wilcoxon signed-ranks test.

**Table 3:** Comparison of the logarithm of minimal angle of resolution best-corrected visual acuity before and after cataract surgery.

Groups	Before cataract surgery	After cataract surgery^*∗*^	*P* value^†^
All the included eyes (*n* = 26)	0.52 ± 0.40	0.20 ± 0.31	<0.001
SRF group (*n* = 12)	0.72 ± 0.44	0.33 ± 0.39	0.003
Non-SRF group (*n* = 14)	0.34 ± 0.26	0.10 ± 0.15	0.001

The data are presented as mean ± standard deviation. SRF = subretinal fluid. ^*∗*^The timing of measurement was averagely 3.6 months in all the included eyes, 3.7 months in the SRF group, and 3.5 months in the non-SRF group. ^†^Statistical analysis was performed using Wilcoxon signed-ranks test.

## Data Availability

The data are available on request (Kim's Eye Hospital IRB).
